# Contributions of Europeans to Xenotransplantation Research: 1. Pig Organ Xenotransplantation

**DOI:** 10.3389/ti.2025.14041

**Published:** 2025-02-27

**Authors:** Zuzanna Iwanczyk, Krish Vasudev, Emanuele Cozzi, David K. C. Cooper

**Affiliations:** ^1^ Department of Surgery, Center for Transplantation Sciences, Massachusetts General Hospital/Harvard Medical School, Boston, MA, United States; ^2^ Transplantation Immunology Unit, University of Padua Hospital, Padua, Italy

**Keywords:** Europeans, organs, pig, USA, xenotransplantation

## Abstract

Xenotransplantation has a rich history, marked by European pioneers who laid the groundwork for many breakthroughs in the field. Pig organ xenotransplantation offers a solution to the global shortage of deceased human donor organs, whilst allowing the modification of the donor graft itself. The field has continued to garner interest, particularly with the recent advent of simpler and faster genetic-engineering technologies. This review highlights the contributions of European researchers to xenotransplantation, spanning pig kidney, heart, liver, and lung transplantation. Research has focused on (i) identifying and deleting key xenoantigens and modifying the source pig by expression of human “protective” proteins and (ii) testing novel immunosuppressive regimens. These contributions have played key roles in advancing xenotransplantation from the laboratory to early clinical experiments. Europeans have also addressed the potential risks of xenozoonotic infections and the regulatory challenges. The research endeavours of groups in Europe are summarized. Several European researchers moved either permanently or temporarily to US institutions, and their insight and innovations are also highlighted. While we aim to recognize the significant contributions of European physicians and scientists in this article, it is not an exhaustive list of all those who have influenced the field.

## Graphical Abstract

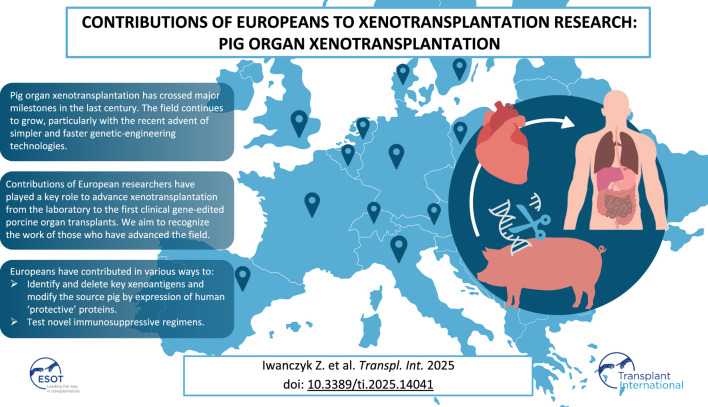

## Introduction

Europeans played a pioneering role in the late 19th and early 20th centuries by transplanting organs from various animals into human recipients - with very poor results [1, 2]. The work of the French experimental surgeon, Alexis Carrel, needs to be highlighted because he and his American colleague, Charles Guthrie, introduced a more reliable technique of anastomosing blood vessels, without which the transplantation of organs would have proved difficult. For this contribution, Carrel was awarded the Nobel Prize for Physiology or Medicine in 1912.

There was then a lull in advances in transplantation until the Ukrainian, Yu Yu Voronoy, carried out a small series of clinical kidney *allo*transplants over several years beginning in the 1930s. However, he did not explore the use of animals as potential sources of organs. Activity in xenotransplantation began again with the work of the American, Keith Reemtsma, who transplanted chimpanzee kidneys into six patients in 1963, one of whom lived for 9 months. This was closely followed by several other American and European surgeons who transplanted kidneys from nonhuman-primates (NHPs) and by James Hardy (USA) and his colleagues who carried out the world’s first clinical heart transplant, again from a chimpanzee. American surgeon, Tom Starzl, soon followed by transplanting livers from NHPs into human recipients, again with little success.

Only one surgeon during this era, the Frenchman Rene Küss, a very early pioneer in kidney allotransplantation, transplanted a kidney from a pig; he was shocked by the rapid speed of rejection - that occurred within minutes.

It was not until the mid-1980s that serious attention turned from NHPs to pigs as the source of organs. There were several logistical reasons for this redirection of attention (Table 1). However, a few years earlier, one European surgeon, Roy Calne, had explored pig liver transplantation in NHP recipients.

**TABLE 1:** The advantages and disadvantages of the pig as a potential source of organs and cells for humans.

Availability	Unlimited, whenever required
Breeding potential	Good
Period to reproductive maturity	4–8 months
Length of pregnancy	114 ± 2 days
Number of offspring	5–12
Growth	Rapid (adult human size within 6 months)^a^
Size of organs for all ages of humans	Adequate
Anatomical similarity to humans	Close
Physiological similarity to humans	Close
Relationship of immune system to humans	Distant
Knowledge of tissue typing	Considerable (in selected herds)
Blood type compatibility with humans	ABO-blood type compatibility can be assured [All pigs will be of type O (non-A)]
Experience with genetic engineering	Considerable
Risk of transfer of infection (xenozoonosis)	Low
Availability of designated pathogen-free pigs	Yes
Cost of maintenance Public opinion	Under the biosecure designated pathogen-free conditions required by the national regulatory authorities the costs will be significant Generally supportive considering that, in the US alone, more than 100 million pigs are slaughtered annually for feeding purposes only

^a^ Various miniature pigs reach a maximum weight of 10%–50% of the weight of domestic pigs.

## Roy Calne (United Kingdom) – Pig Liver Transplants in NHPs

The late Roy Calne, renowned for his work in clinical allotransplantation, left a legacy of firsts within his field [3]. He is most remembered for the introduction of immunosuppressive therapy, notably including azathioprine, cyclosporine, rapamycin, and alemtuzumab (Campath). Working at the University of Cambridge, he also carried out the first liver transplant in Europe in 1968, the world’s first combined liver-heart-lung transplant in 1987 (with cardiothoracic surgeon John Wallwork), and the first intestinal transplant in the United Kingdom. However, Calne also contributed to early work in xenotransplantation by carrying out a small series of pig liver transplants in NHP recipients in the late 1960s [4, 5]. The results were again disappointing.

Some years later, Calne suggested the terms “concordant” and “discordant” xenotransplants to distinguish between those rejected relatively slowly by a cellular mechanism (similar to allotransplantation) and those rejected hyperacutely by an antibody-dependent complement-mediated mechanism (similar to that seen in ABO-incompatibility or in HLA-sensitized recipients) [6].

## The Next Step

In the early 1980s, one of the present authors (David Cooper [United Kingdom]), while working at the University of Cape Town, carried out a study on xenotransplantation between different species of NHPs (a concordant model) [7]. In 1985, he and another European, Gerfried Lexer [Austria], established what has become a standard experimental model in xenotransplantation – the pig-to-NHP heterotopic heart transplant model (a discordant model), in which the heart is anastomosed to vessels in the recipient’s abdomen or neck but is not contributing to the support of the circulation [8, 9]. They confirmed that rapid rejection of a pig graft (hyperacute rejection) occurred and was a result of the binding of human serum anti-pig antibodies to the graft and the activation of the complement cascade. Treatment with the available immunosuppressive drugs had little or no effect in delaying rejection [9].

They introduced the concept of perfusing one organ from the pig, e.g., the kidney, to adsorb anti-pig antibodies from the potential recipient’s blood before transplanting another organ, e.g., the heart, from the same pig.

## Guy Alexandre (Belgium) – Pig Kidney Transplants in Baboons

The late Guy Alexandre (Catholic University of Louvain) took it one step further by transplanting a life-supporting pig kidney intra-abdominally and removing the recipient’s native kidneys [10]. Like Calne, Alexandre was an early contributor to organ allotransplantation [11]. In 1963, he was the first to suggest and implement the use of kidneys from brain-dead donors (i.e., before circulatory death), a notion that was very controversial at the time. He went on to introduce methods to safely transplant ABO-incompatible kidneys by prior removal of anti-AB antibodies by plasmapheresis. These two major innovations, the use of organs from brain-dead donors and ABO-incompatible organ transplantation, have done a great deal to increase the donor pool of allografts and, in our opinion, Alexandre did not receive sufficient recognition for these contributions. For example, he was not awarded the prestigious Medawar Prize of The Transplantation Society.

However, in addition, he was a very early contributor to research into xenotransplantation. Using his experience of clinical ABO-incompatible allotransplantation, he utilized plasmapheresis to remove anti-pig antibodies from the NHP recipient before pig kidney transplantation. He achieved the longest survival time of baboons with a pig kidney at the time (22 days) in 1989 [10]. Unfortunately, although ‘accommodation’ developed after many ABO-incompatible allotransplants (in which the return of anti-AB antibodies was *not* followed by rejection), it did not occur after xenotransplantation. Although xenograft survival was short compared with that which we achieve today, Alexandre’s work undoubtedly played a role in advancing xenotransplantation.

## The Imutran (United Kingdom) Group

In the mid-1980s after the introduction of cyclosporine as an immunosuppressant, the number of patients waiting for a transplant significantly increased world-wide and it became apparent that there was a growing need for an alternative source of organs for patients with terminal organ failure.

In this context, xenotransplantation was viewed by some as one of the possible approaches to explore, although the immunological barriers to successful xenotransplantation, in particular, hyperacute rejection, were major obstacles to overcome. Scientists at that time estimated that there were two approaches to overcome this immunological barrier. The first consisted in the development of new molecules or immunotherapies to enable the recipients to accept the xenograft. The second was to “manipulate” the pig organ to render it more compatible with the recipient.

Among those greatly interested in finding a solution to the shortage of human organs and render xenotransplantation a viable option was University of Cambridge transplant immunologist, David White, who was well-known in the transplantation community for his contributions, with Roy Calne, to the development and clinical introduction of cyclosporine. White immediately understood that working on the donor (as opposed to the recipient) was the road to pursue. He used the largely unrecognized fact (at that time) that complement-regulatory proteins are species-specific (and prevent autologous complement from injuring native cells), to explore whether the transgenic expression of a human complement-regulatory protein in pigs would protect the pig vascular endothelial cells from injury by human complement [12]. (Augustin Dalmasso in the USA had the same idea [13].).

In the early 1990s, White, together with his surgical colleague, John Wallwork, and a local businessman, founded Imutran, a biotech company based in Cambridge whose key objective was to pave the way to clinical xenotransplantation [12, 14, 15]. The researchers were mainly British but with a number of foreign team members. Among the most important were molecular biologists Nikos Yannoutsos (Greece) and Gillian Langford (United Kingdom), and Richard Lancaster (United Kingdom) who had expertise in the generation of transgenic pigs by pronuclear microinjection of fertilized oocytes with purified DNA [16–18].

The initial *in vitro* experiments conducted by White’s team (that included one of the present authors [Emanuele Cozzi, Italy]), and by other groups in the United States, showed that murine cells expressing a human complement inhibitor, such as human decay-accelerating factor (hDAF), were resistant to human complement injury. Such experiments led to the generation of transgenic mice expressing hDAF in various tissues whose cells were resistant to human serum complement-mediated lysis [19]. These convincing results suggested that an appropriate gene-edited pig might possibly represent an unlimited source of organs for clinical transplantation and encouraged Imutran and other companies (such as Nextran and Alexion, both in the United States) to invest considerable resources in this specific area of research. Indeed, the competition at that time was severe. These companies received funding from “big pharma” companies already involved in the healthcare sector. In particular, Imutran received funding from Sandoz (that soon merged with Ciba-Geigy to become Novartis) while Nextran was supported by Baxter.

Thanks to the support provided by Sandoz/Novartis, Imutran could strengthen its infrastructure, reinforce its manpower and logistics, and initiate an intense research activity aimed at genetic engineering pigs as a possible source of organs for clinical purposes. To this end, Imutran appointed a team of molecular biologists with expertise in gene-editing of cells and made the necessary investments to enable the opening and maintenance of facilities necessary for the generation and breeding of genetically-engineered pigs. The results were promising with the successful birth of the first hDAF gene-edited pig litter during the final days of 1992, an achievement that opened the way to fundamental proof-of-concept studies.

As a first step, *ex-vivo* perfusion studies demonstrated that organs from hDAF transgenic pigs were indeed resistant to human complement-mediated injury. Subsequently, hDAF pig hearts were transplanted heterotopically into non-immunosuppressed NHPs and proved to be resistant to hyperacute rejection, with a median survival of 5.1 days. In immunosuppressed NHPs the hearts survived for up to 62 days, an accomplishment that enabled the initiation of life-supporting studies in NHPs.

Such encouraging results boosted the visibility of Imutran and encouraged Novartis to purchase the company. However, they soon also attracted the attention of ‘animal rights’ activists in the United Kingdom which was one factor - together with concern of the medicolegal risks of the transfer of porcine endogenous retroviruses (PERVs) to a patient with a pig graft - that eventually persuaded Novartis to shut down Imutran and transfer its know-how to a newly created Boston US-based company, Immerge.

Before its demise, Imutran contributed much to advance pig heart and kidney xenotransplantation, and also carried out pig liver transplantation with a Spanish team [20]. It also generously provided some of its hDAF pigs to centers in the United States, which did much to stimulate the entire field of xenotransplantation research. In summary, Imutran’s work provided the first step towards the use of gene-edited pigs as sources of organs for clinical transplantation.

## Early Work in Denmark

During the 1980s and 1990s, Ejvind Kemp and his colleagues in Odense and Copenhagen were very active in various fields of xenotransplantation research, ranging from rodent studies [21] to the histopathology of rejection [22] to large animal experiments [23]. Ejvind Kemp’s great interest in this new field of research is exemplified by his authorship of a book on xenotransplantation as early as 1978 [24].

## Glycobiology and Clinical Experiments in Sweden

Swedish groups proved very active in the early days of xenotransplantation research, especially in islet transplantation (see Bottino et al, this issue) in part because of excellent collaboration between their major medical universities. Swedish surgeon/scientist, Michael Breimer (initially with Bo Samuelsson) at Gothenborg University, was active in the field of glycobiology in relation to pig organ xenotransplantation. He characterized the structure and tissue distribution of carbohydrate xenoantigens from several organs and tissues from various wild-type and gene edited pigs [25–27].

In addition, after discussion with Ken Welsh, David Taube, and others in the United Kingdom [28], the Swedish team carried out two bold experiments in 1996 [27, 29, 30]. With the consent and cooperation of two patients undergoing regular dialysis, they inserted a wild-type pig kidney into the circuit so that the kidney was perfused with the patient’s blood. Previous plasmapheresis had been carried out to deplete anti-pig antibodies and no immunosuppressive therapy was administered. In one patient, the kidney survived for 65 min before being rejected. The second patient developed hemodynamic shock within 15 min, presumed to be associated with release of cytokine and complement factors, necessitating termination of the experiment.

Hakan Widner and his colleagues at the University of Lund were very active in the field of embryonic fetal pig brain tissue transplantation to cure neurodegenerative conditions [31].

## The Work of Europeans in the United States

David Cooper relocated to the United States where he worked at various centers and collaborated with several European scientists – some temporarily or permanently resident in the USA and others based in Europe. He participated in the mentoring of several young physicians and surgeons who went to the USA to gain experience in xenotransplantation. Several of these young men and women made major contributions and some have since become leaders in the field of transplantation.

### Oklahoma Transplantation Institute and Oklahoma Medical Research Foundation (OMRF)

Initially based in Oklahoma City, Cooper’s group collaborated with a small Canadian biotech company (Chembiomed, based in Edmonton) to identify the major carbohydrate pig antigen against which humans (and NHPs) have natural (preformed) antibodies that initiate hyperacute rejection. Based on what was known about ABO blood group antigens (which are oligosaccharides), the Oklahoma team reasoned that anti-pig antibodies may also be directed to pig carbohydrate antigens. This proved correct. A key and very important member of the Oklahoma team, Eugene Koren, an OMRF scientist from Croatia, was responsible for isolating the antibodies that bind to pig vascular endothelial cells. These were then tested in Edmonton against a panel of synthetic oligosaccharides and found to bind to galactose-α1,3-galactose (Gal) (Table 2) [32, 33]. This was a major step forward and confirmed an observation (hitherto unknown to the Oklahoma team) made earlier by Uri Galili (from Israel).

**TABLE 2:** Carbohydrate xenoantigens that have been deleted in genetically-engineered pigs.

Carbohydrate (abbreviation)	Responsible enzyme	Gene-knockout pig
1. Galactose-α1,3-galactose (Gal)	α1,3-galactosyltransferase	GTKO
2. N-glycolylneuraminic acid (Neu5Gc)	Cytidine monophosphate-N-acetylneuraminic acid hydroxylase (CMAH)	CMAH-KO
3. Sda	β-1,4N-acetylgalactosaminyltransferase	β4GalNT2-KO

Their original plan was to deplete the human serum of anti-Gal antibodies by immunoadsorption on columns of synthetic Gal glycans but, during discussion with another close collaborator, Paris-based immuno-geneticist Rafael Oriol, it was determined that the ultimate solution would be to delete expression of Gal in the pig [34]. The technology to do this had not yet been developed and so it was more than a decade before α1,3-galactosyltransferase gene-knockout (GTKO) pigs became available [35, 36].

In the meantime, the team developed methods of immunoadsorption to deplete baboons of anti-Gal antibodies. Marek Niekrasz (Poland) was one of the veterinary surgeons who participated fully in these challenging endeavors. Anti-Gal antibodies were successfully removed from the serum but, despite immunosuppressive therapy, returned within a few days, at which time rejection occurred. There was some collaboration with Robert Rieben’s team (Switzerland) [37], and with a group in the United Kingdom (David Taube, Tom Cairns, Ken Welsh) who were also pursuing this approach, and members of this group visited Oklahoma City to participate in these experiments [28, 38].

Gene Koren led a small and productive subgroup of the team (that at times included several other Croatians) to carry out numerous *in vitro* studies that identified IgM, IgG, IgA, and IgE anti-Gal antibodies [39–42]. Back in Paris, Rafael Oriol investigated numerous animal species for expression of Gal [43, 44].

### Identification of N-Glycolylneuraminic Acid as a Second Pig Carbohydrate Xenoantigen

While these studies were taking place in the USA, a French group identified N-glycolylneuraminic acid (Neu5Gc) as a second pig xenoantigen (Table 2) [45], but this observation was not pursued at that time. It was not until Alex Zhu confirmed this finding in 2002 that interest in Neu5Gc as a xenoantigen began to expand [46].

### Massachusetts General Hospital (MGH)/Harvard Medical School

After approximately 9 years, the Oklahoma team split up, and Cooper accepted an appointment at the Transplantation Biology Research Center (headed by David Sachs) at the MGH. With David Sachs, they made a sustained effort to induce a state of immunological tolerance to pig organs in NHPs, but this proved too great a challenge with the therapies available at the time. The team explored the effect of various immunosuppressive drugs on the “rebound” of anti-pig antibodies after immunoadsorption [47]. Key European participants included Denis Lambrigts (Belgium) and his wife Denise van Calster (Belgium), who arrived at a critical time and did much to improve the productivity of the xenotransplantation program, Leo Buhler (Switzerland), Ian Alwayn and Frank Dor (both from surgeon Jan Ijzermans’ group at Erasmus University in Rotterdam in Netherlands). Frank Dor’s major interest at MGH was the induction of tolerance by spleen allotransplantation, but he was involved in all of the xenotransplantation work.

It was then that another major observation was made. In their studies of pig hematopoietic progenitor cell (HPC) transplantation as a potential means of inducing tolerance to pig xenoantigens, Leo Buhler observed that, despite cyclosporine-based immunosuppressive therapy, the infusion of pig HPCs was followed by sensitization, resulting in a great increase in the titers of anti-pig IgM and, particularly, IgG [48]. Knowing of the introduction of new agents that caused blockade of the T cell co-stimulation pathway in allotransplantation, Buhler found that treatment with an anti-CD154 mAb (rather than cyclosporine) prevented sensitization [48]. This proved to be a major advance in xenotransplantation and, since that time (2000), all truly successful immunosuppressive regimens administered to NHPs with pig organ or cell grafts have been based on CD40/CD154 co-stimulation pathway blockade.

Coagulation dysfunction had been predicted by several physicians interested in xenotransplantation research, including Simon Robson [United Kingdom and South Africa, by then based in Boston in the USA, where he was joined by a series of European research fellows, e.g., Matz Schmelze (Germany), Jan Schulte am Esch (Germany), Christoph Kopp (Austria), and Daniel Candinas (Switzerland)]. However, it was at the MGH that several fundamental observations were made from the pig-to-NHP studies progressing at that time. Key observations were made by Thomas Kozlowski (Poland), Frank Ierino (Australia), and Leo Bühler who independently observed that rejection of a pig organ was accompanied by a thrombotic microangiopathy in the graft and a disseminated coagulopathy in the recipient [49, 50]. Ian Alwayn (Netherlands), working partly with Simon Robson, made several significant contributions to our understanding of these phenomena [51–54].

When GTKO pigs were born at Immerge in Boston [36], the team progressed to the transplantation of their organs into NHPs, using an anti-CD154mAb-based immunosuppressive regimen (reviewed by Sanatkar et al in this issue). Hendrik-Jan “Henk” Schuurman (Netherlands), then the vice president for research at Immerge, played a leading role in the production of these pigs. Heterotopic heart transplantation was associated with longer function than reported previously, with maximal survival of 6 months [55]. Maximal survival of baboons with a life-supporting GTKO pig kidney graft was approximately 3 months [56].

Christoph Knosalla (Germany) and Bernd Gollackner (Austria) joined the team. Christoph continued the GTKO pig heart transplant studies but faced challenging problems with coagulation dysfunction [57, 58]. Bernd carried out some valuable *in vitro* studies with Simon Robson [59, 60], Nicolas Mueller (Switzerland) and Jay Fishman [61–63].

Despite the success that Immerge had achieved, Novartis withdrew its financial support, just as it had withdrawn support for Imutran a few years earlier. When Immerge was forced to close, the core of the xenotransplant research team was invited (by Tom Starz) to relocate to Pittsburgh, where the University of Pittsburgh Medical Center (UPMC, the company that owned several hospitals in the region) had recently purchased the small biotech company, Revivicor (Blacksburg, VA), that had produced the first GTKO pigs a few weeks before Immerge. (Revivicor was the remnant of the Edinburgh [United Kingdom] company PPL Therapeutics that had successfully cloned the first large mammal - Dolly, the sheep [64].)

### University of Pittsburgh

Jan Ijzermans’ support of the xenotransplantation research program continued with the arrival of Pleunie Rood, and subsequently Dirk van der Windt and Eefje Dons from Rotterdam. Although Pleunie Rood’s main contributions were related to pig islet transplantation in NHPs (see Bottino et al in this issue), she, together with other research fellows, carried out valuable *in vitro* studies determining the extent of anti-pig antibody binding to GTKO pig cells [65, 66]. Antibody binding was greatly reduced when Gal was not expressed on the pig cells.

Rood, and later Dons, carried out some challenging studies in infant baboons and demonstrated that treatment with an anti-CD154mAb inhibited the production of natural (preformed) antibodies [66, 67]. As natural antibodies (including both anti-ABO and anti-pig) had been considered to be T cell-independent, their data questioned this conclusion. If treatment was initiated soon after birth of the baboon (before anti-nonGal antibodies had begun to be produced), this resulted in baboons that developed *no* serum anti-GTKO pig antibodies until several weeks after cessation of anti-CD154mAb therapy [67]. This opened the possibility of carrying out pig organ transplants in human infants in the absence of anti-pig antibodies. This could include, for example, babies with life-threatening congenital heart disease for which no other therapy offered much chance of success (see below).

Working with Rita Bottino (Italy), Dirk van der Windt also concentrated much of his attention on islet xenotransplantation (see Bottino et al in this issue), but he also investigated alemtuzumab as an immunosuppressive agent in NHPs [68].

Later, GTKO pigs that expressed human complement-and/or coagulation-regulatory proteins became available from Revivicor. In collaboration with the late Agnes Azimzadeh (France), organs from these pigs were demonstrated to protect them further from the human (or NHP) immune response [69]. By this time, however, because the anti-CD154mAbs had been demonstrated to be thrombogenic [70], immunosuppression was achieved with an anti-CD40mAb, first introduced into xenotransplantation by Mohiuddin et al [71], but which proved not as effective as an anti-CD154mAb.

Burcin Ekser (born in Turkey and trained in Italy), a very productive Pittsburgh research fellow, working with liver transplant surgeon Bruno Gridelli (Italy, who had been active in xenotransplant research for some time), carried out some important studies of GTKO pig liver transplantation in NHPs [72, 73]. They demonstrated very clearly that within minutes a profound thrombocytopenia developed in the recipient which did not recover and ultimately resulted in internal bleeding, requiring euthanasia [73]. However, there was evidence of quite good function of the liver [72], suggesting that there was a potential for a pig liver to support a patient at least as a bridge until a liver from a deceased human donor became available.

### University of Alabama at Birmingham

**FIGURE 1:**
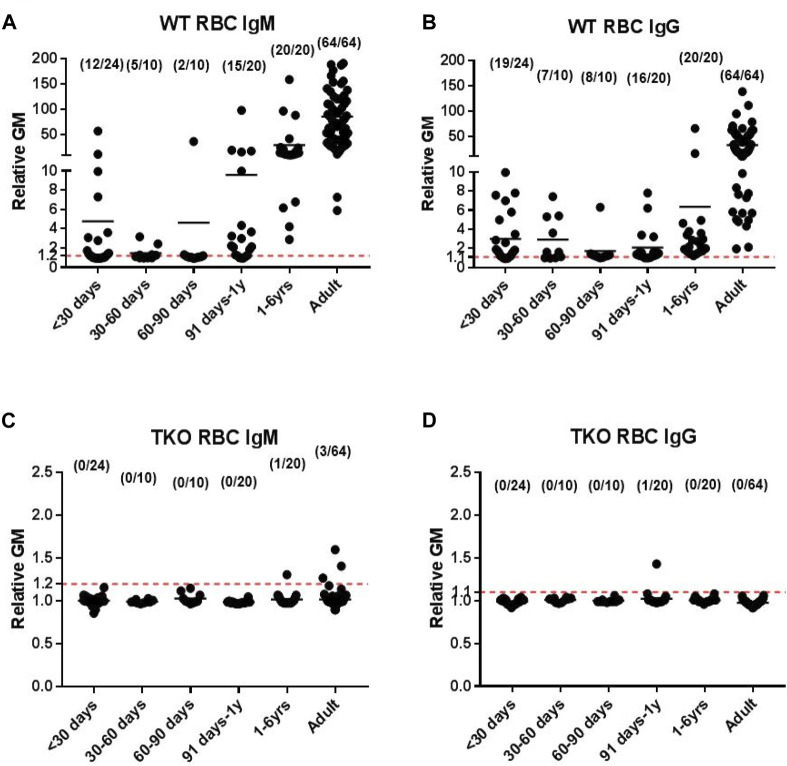
Human serum IgM and IgG antibody binding to wild-type (WT) pig red blood cells (RBCs) (**A, B**, respectively) and triple-knockout (TKO) pig RBCs **(C, D)** at different ages. (Note the difference in the y-axis between binding to WT and TKO RBCs.) (Reproduced with permission from Li Q et al. Ann Thorac Surg. 2020; 109:1,268–1,273).

Enticed by the fact that Revivicor (by now owned by United Therapeutics) was building a ‘clean’ (designated pathogen-free) pig facility in Birmingham, Alabama, that would enable clinical pig organ xenotransplantation to proceed, members of the (by now “nomadic”) research team relocated to Birmingham, where Joe Tector was also establishing a research program. Triple-knockout (TKO) pigs became available from Revivicor (Figure 1) but, despite also expressing six human transgenes and therapy with an anti-CD40mAb, the results of pig organ transplants remained mixed, in part due to the fact that all Old World NHPs have natural antibodies against these cells [74, 75].

Nevertheless, based on previous studies by this group (Figure 1) [66, 67, 76, 77], encouraging results of TKO pig orthotopic heart transplantation were obtained in collaboration with Birmingham pediatric cardiac surgeon David Cleveland, with survival of one baboon for almost 8 months [78, 79].

Sadly, when Devin Eckhoff, who had been active in establishing the xenotransplantation research program at UAB, moved to Boston, problems arose, and the team disbanded. Cooper moved back to the MGH where he continues an active research program today. An immunosuppressive regimen based on an anti-CD154mAb combined with rapamycin appears to prevent rejection in the TKO pig-to-baboon model [80].

Throughout these past 35 years intermittent efforts were also made to investigate the possibility of pig red blood cells being used for clinical transfusion. Under the mentorship of Frank Dor and other research fellows, visiting European students, such as Foad Rouhani (United Kingdom), played a significant role in these studies [81].

## Agnes Azimzadeh and Lars Burdorf – Pig Lung Transplantation

As members of Robin Pierson’s group, initially at Vanderbilt University, then at the University of Maryland, and subsequently at MGH, two Europeans, Agnes Azimzadeh (France) and Lars Burdorf (Germany), contributed very significantly to intensive studies of pig-to-baboon lung transplantation (as well as to many other studies). This model has been found to be extremely challenging and progress has been slow, with graft survival still frequently measured in days rather than weeks [82]. Nevertheless, this group’s dedicated efforts have made inroads in identifying the extreme immunological problems that prevent progress. Agnes Azimzadeh, in particular, also contributed to numerous other studies in xenotransplantation [83].

Several other Europeans (most commonly from Germany or France) were members of this group over the years, for example, Carsten Schroeder (Germany), with the most recent being Franzi Pollok (Germany), Ryan Chaban (Germany) [84], and Sara De Taeye (Belgium).

## Christopher McGregor (United Kingdom) – Pig Heart Transplantation

Chris McGregor, a British cardiothoracic surgeon and researcher, has for many years divided his time between the USA and United Kingdom. His initial research was largely directed towards pig heart xenotransplantation in NHPs and he collaborated with the U.S. biotech company, Nextran, whose research was largely directed by John Logan, also of Scottish origin. When at the Mayo Clinic, McGregor tested induction therapy with ATG and rituximab and maintenance therapy that included rapamycin in a tacrolimus-based immunosuppressive regimen; mean heart graft survival was extended longer than reported previously [85, 86]. Importantly, his close and long-term colleague, Guerard Byrne, identified the glycan Sda as the third carbohydrate xenoantigen against which humans have natural antibodies [87]. Its identification enabled triple-knockout pigs to be produced (Table 2), initially by Estrada et al. [74], moving xenotransplantation much closer to clinical experiments.

In recent years, McGregor and Byrne have concentrated their attention on testing gene-edited pig cardiac valve bioprostheses and established a pig-to-sheep model in Europe to do this [88]. These bioprostheses, at present based on GTKO pigs, should delay the deterioration seen in current pig cardiac valve bioprostheses after implantation in young patients.

## The Munich Group

A further group that has made major contributions to organ xenotransplantation is that from Ludwig Maximilians University in Munich, Germany. The early work of Claus Hammer (1940–2015), who held degrees in both medicine and veterinary medicine, related to the anatomical, physiological, and biochemical aspects of xenotransplantation, on which topic he was the world authority [89]. He also carried out interesting studies of concordant xenotransplantation, e.g., between fox and dog [90], and proved himself to be one of the most productive researchers in the 1980s and 1990s.

A new research endeavour at the university was established formally in 1998 by cardiothoracic surgeon, Bruno Reichart, to develop successful pig-to-baboon heart transplantation. It took several years for success to be achieved [91]. Difficulty in producing GTKO pigs was overcome by collaboration with Revivicor, but in recent years the Munich group has been a leader in the field. Its ‘breakthrough’ moment came in 2018 when it overcame the early pig cardiac graft failure that had prevented progress for several years [92], initially described and investigated by Chris McGregor’s group [93]. This was suspected not to be associated with rejection; the data suggested it was due to a sensitivity of the pig heart to ischemia-reperfusion injury.

By using the Steen system of myocardial protection (a Swedish advance) [94], the problem was resolved and prolonged function (3–6 months) after pig orthotopic heart transplantation in baboons was consistently achieved for the first time [92]. (It appears, however, that, when the pig heart is small and the ischemic period is short, adequate myocardial protection can be achieved by static cold storage after cardioplegia with del Nido solution, which is much simpler and less expensive [79].)

Much of the success of the Munich group has been due to the work of Eckhard Wolf, an expert on gene-editing in pigs, who has also developed disease models for conditions such as diabetes mellitus and rare monogenic diseases [95, 96]. His work in tailored medical models in mice and pigs has led to innovative models to not only address the organ shortage but also improve treatment options for various diseases.

One innovation from this group was to demonstrate that, when the pig organ grew quickly after transplantation, thus leading to some compression within the NHP recipient, jeopardizing its function, deletion of the gene for growth hormone receptors reduced growth [97]. This problem, of course, has not been seen when the organs are taken from a miniature breed of pig.

## Hannover Medical School and Friedrich-Loeffler Institute

A second group in Germany has been very active in the production of gene-edited pigs for xenotransplantation (and for several other reasons, e.g., as models of diseases), but less active in collaborating with surgical groups to transplant organs from these pigs in NHPs. This group, established in the 1990s and headed by veterinary scientist Heiner Niemann, and including well-known colleagues such as Bjorn Petersen and Reinhard Schwinzer, has contributed many innovations to the field over many years [98–102]. It has had a special interest in the introduction of ‘anti-inflammatory’ transgenes [103, 104].

## Parallel Studies in Xenotransplant Immunology

In parallel with these time-consuming and expensive studies in the pig-to-NHP model, several European laboratories were investigating aspects of xenotransplantation immunology by *in vitro* assays. Space does not allow a detailed discussion of their wide-ranging studies, but they included groups in the United Kingdom (London, led by Anthony Dorling and Robert Lechler) [105, 106], France (Nantes, Jean-Paul Soulillou) [107], Switzerland (Jorg Seebach, Geneva/Zurich, and Robert Rieben, Bern) (see this issue). The Nantes group led by Gilles Blancho, also carried out studies in the pig-to-NHP model [108, 109]. Others include Eelco Bouwman (Netherlands), Beverly Hunt (United Kingdom), Luca Inverardi (Italy), Ignazio Marino (Italy), Ruggero Pardi (Italy), Leendert Paul (1946–2004, Netherlands and Canada), Raymond Reding (Belgium), Marlene Rose (United Kingdom), and Karen Ulrichs (Germany), among others.

## The Potential Risk of Xenozoonosis

The potential risk of xenozoonosis has received much attention by experts in the field, and European infectious disease experts have played important roles. Joachim Denner (Germany) is one of the world experts on PERVs and has developed highly sensitive methods to detect potential xenozoonotic porcine viruses (see Denner J in this issue). His impact on virology and biomedical research has advanced knowledge on the safety of xenotransplantation. Others include Linda Scobie (United Kingdom) and Nicolas Mueller (Switzerland) (see Scobie & Mueller in this issue), Ralf Tonjes (Germany) (see Tonjes in this issue), and the late Henk Schuurman (Netherlands). Henk Schuurman, who spent much of his career commuting between Europe and the USA, put his knowledge into practice by overseeing the construction and functioning of the Spring Point project in Wisconsin, a facility built to breed and house pigs under designated pathogen-free conditions that would be acceptable to the US FDA [15].

In addition, Ralf and Henk played leading roles in advising participants and prospective participants on the regulatory aspects of xenotransplantation. Henk was perhaps unique in having an in-depth knowledge of regulation on both sides of the Atlantic. The regulatory landscape poses stringent requirements to mitigate the potential risks associated with the clinical use of xenografts, most notably the risk of xeno-zoonotic pathogen transmission [110]. Ralf and Henk have aided in navigating these regulations to create guidelines for clinicians and pave the way towards the development of safe and realistic xenotransplantation therapies [111, 112].

## Comment

We thus conclude that over the past 50 years European surgeons and scientists have contributed significantly to progress in xenotransplantation research. Space has not allowed us to draw attention to all of the Europeans who have worked in this field, and we apologize for our omissions. We also apologize to the many non-Europeans who have contributed to the research reviewed in this article but who have not been identified.

## Abbreviations

Gal, galactose-α1,3-galactose; GTKO, α1,3-galactosyltransferase gene-knockout; hDAF, human decay-accelerating factor; NHP, nonhuman primate.
